# Hedy: a groundbreaking revelation of cartilage oxygen homeostasis

**DOI:** 10.3724/abbs.2024116

**Published:** 2024-07-22

**Authors:** Haoliang Hu, Labapuchi, Kerui Huang, Yanling Long, Linxi Chen

**Affiliations:** 1 College of Medicine Hunan University of Arts and Science Changde China; 2 Institute of Pharmacy and Pharmacology College of Basic Medical Science Hengyang Medical School University of South China Hengyang China; 3 The Second Affiliated Hospital Department of Pain and rehabilitation Hengyang Medical School University of South China Hengyang China

Oxygen plays a vital role in cellular respiration within the human body, providing essential energy for life sustenance and serving as a key component in important biochemical reactions necessary for cellular homeostasis and maintaining cellular viability. In human erythrocytes, hemoglobin (Hb) is central to the process of binding with and releasing oxygen, thereby regulating systemic oxygenation and contributing significantly to physiological homeostasis [1]. While oxygen supply and adaptation mechanisms are well understood in vascularized tissues, avascular tissues present unique challenges. Recent studies have shed light on the multifaceted roles of Hb in avascular tissues, such as cartilage tissue, which extend beyond the traditional functions of Hb in the circulatory system [ 2, 3] .

The body responds to tissue hypoxia by activating the hypoxia-inducible factor (HIF) signaling pathway, adjusting erythropoiesis, and regulating vascular constriction and dilation, leading to complex physiological reactions [4]. These mechanisms work together to maintain tissue oxygen homeostasis, counteracting adverse effects and demonstrating remarkable adaptability to low-oxygen environments. Moreover, oxygen diffusion from red blood cells (RBCs) is particularly inefficient in avascular or highly oxygen-consuming tissues, leading to the development of additional mechanisms for rapid oxygen acquisition. For instance, in neurons, which are highly oxygen-demanding cells, neuroglobin stores oxygen and shields the brain from ischemic injury [5]. Similarly, myoglobin, which is abundant in muscle cells, binds with and stores oxygen, ensuring timely and sustained oxygen supply during physical exertion.

Traditionally, Hb is known to play a role in oxygen and carbon dioxide transport within RBCs [1]. Recent research has shown that Hb is also present in nonvascular cells, including alveolar epithelial cells, macrophages, mesangial cells, midbrain dopamine neurons and glial cells, retinal pigment epithelium, and malignant breast tumor cells [ 3, 6] . In these nonvascular cells, Hb performs various unconventional biological functions, such as intracellular oxygen transport, oxygen sensing, cellular protection, antioxidant defense, and adaptation to low oxygen conditions. In addition, abundant Hb bodies, which act as oxygen carriers to maintain intracellular oxygen homeostasis and adapt to diverse hypoxic stressors, have been discovered within chondrocytes [2].

Cartilage, which is predominantly found in weight-bearing regions, particularly joint surfaces, is crucial for providing structural support, protection, and lubrication to joints. As a component of avascular connective tissue, chondrocytes have a unique way of obtaining oxygen [2]. Oxygen penetrates the cartilage matrix, which is made of collagen, proteoglycans, and water, to reach chondrocytes and meet their oxygen needs. The structural components of cartilage, such as collagen and proteoglycans, significantly affect the dynamics within the cartilage matrix. Moreover, mechanical stress on cartilage tissue, which is influenced by factors such as movement and weight-bearing, slows oxygen diffusion, thereby worsening hypoxic conditions.

**Figure 1:**
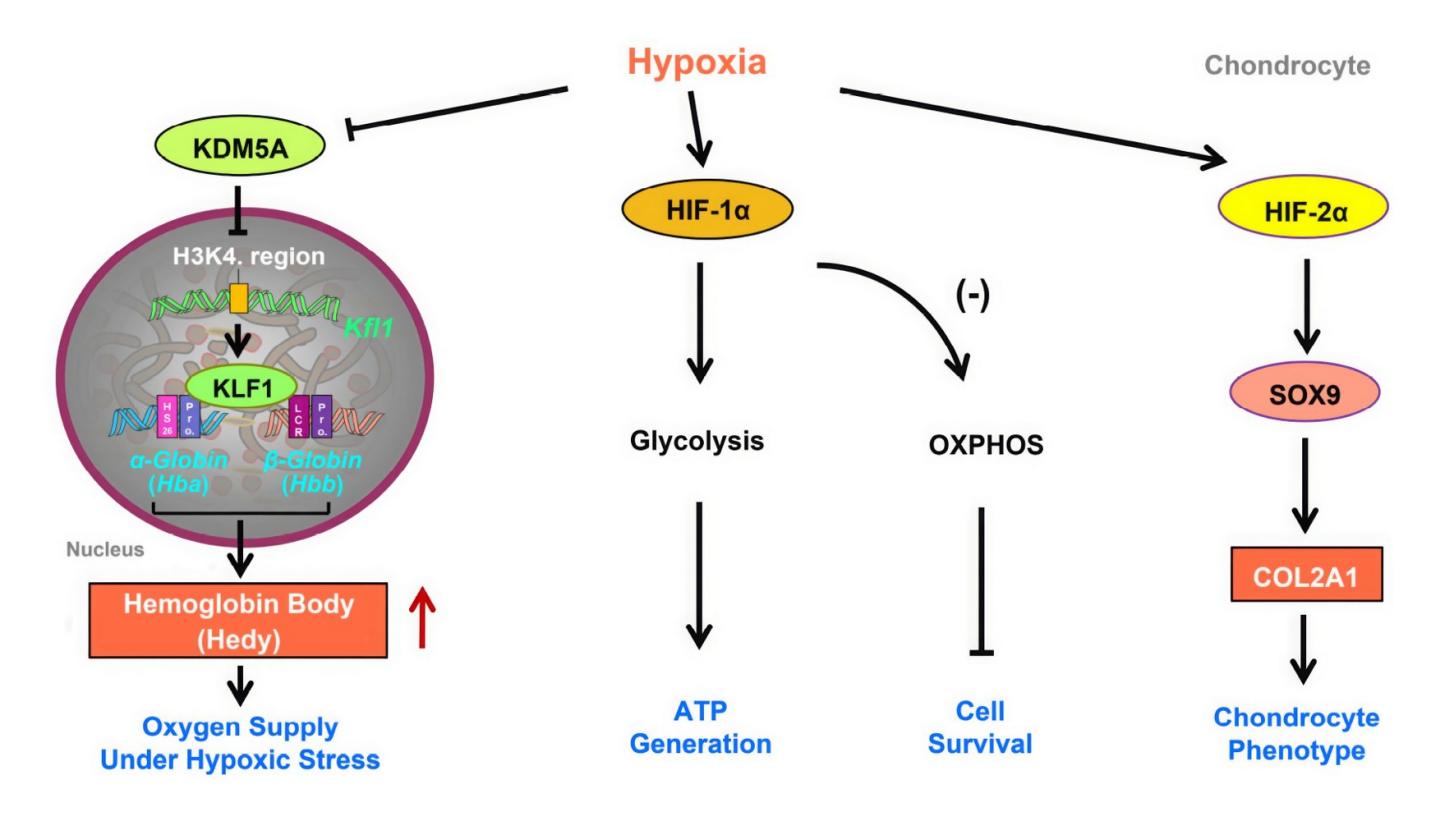
Figure 1 Schematic diagram of the adaptive responses of chondrocytes to hypoxic conditions Conventionally, chondrocyte hypoxia activates the HIF signaling pathway primarily via the upregulation of HIF-1α and HIF-2α levels. HIF-1α enhances glycolytic processes in chondrocytes, resulting in elevated ATP production to support essential metabolic functions. Deletion of HIF1α induces mitochondrial oxidative phosphorylation (OSPHOX) in chondrocytes, which leads to increased oxygen consumption and severe hypoxia and ultimately compromises cell survival. Under hypoxic conditions, HIF-2α enhances the expression of the cartilage transcription factor SOX9 to facilitate the synthesis of key cartilage matrix components such as COL2A1. This process promotes extracellular matrix synthesis and maintains the chondrocyte phenotype. Hypoxia induces hemoglobin body (Hedy) production in chondrocytes via a KLF1-dependent mechanism rather than through the HIF signaling pathway, contributing to an internal oxygen supply for chondrocytes under hypoxic conditions. In particular, hypoxia-mediated inactivation of the JmjC histone demethylase KDM5A facilitates epigenetic upregulation of Klf1 via enhanced H3K4me3 modifications.

In chondrocytes, hypoxic induction of the HIF signaling pathway involves various mechanisms to adapt to hypoxic environments ( Figure 1). This pathway regulates chondrocyte adaptation to hypoxic stress via increased levels of HIF-1 and HIF-2. Notably, HIF-1α plays a crucial role in metabolic reprogramming by promoting nonoxidative glycolysis and suppressing mitochondrial respiration. HIF-1 mediates cellular adaptation to hypoxia by generating ATP from nonoxidative glycolysis through the activation of glycolytic genes [ 7, 8] . However, when the gene encoding HIF-1α is deleted, chondrocytes switch to mitochondrial oxidative phosphorylation, consume excessive oxygen, and cause severe hypoxia, ultimately impairing chondrocyte viability. In addition to HIF-1α, HIF-2α also contributes to the hypoxic regulation of chondrocytes. HIF-2α is essential for hypoxic induction of the differentiated chondrocyte phenotype. In particular, HIF-2α enhances the synthesis of cartilage matrix and maintains the chondrocyte phenotype by regulating the expression of specific factors such as SOX9 [9]. Therefore, HIF-2α plays a critical role in regulating the expressions of cartilage matrix genes such as *COL2A1* and *aggrecan*, which are essential for chondrocyte function. Further research is necessary to fully comprehend the specific mechanisms through which chondrocytes maintain oxygen homeostasis.

In a recent breakthrough, Zhang’s team [2] identified an Hb body termed “Hedy” within chondrocytes of cartilaginous tissue, marking the first discovery of its kind. This structure shares a morphological resemblance to RBCs from bone marrow, with its formation intricately regulated by hypoxic conditions. The abundant Hb in these chondrocytes consists mainly of hemoglobin subunit β (HBB), with a minor presence of hemoglobin subunit α (HBA), alongside a distinct eosin-positive structure within the cytoplasm. Notably, during early embryonic development (E14.5), there is significant and exclusive expressions of ζ-globin, εy-globin, and β _h1_-globin in growth plate chondrocytes, resembling the phenomenon of globin switching observed in erythroid development [2]. The Hedy structure, which is a membrane-less condensate within the chondrocyte cytoplasm, undergoes liquid-liquid phase separation. Functioning as a localized oxygen reservoir, intracellular Hedy plays a crucial role in chondrocyte adaptation to hypoxia and preservation of fetal cartilage survival.

Hypoxia consistently induces Hb expression in primary chondrocytes, leading to increased levels of *Hba* and *Hbb*. Deletion of *Hif1a* in chondrocytes results in widespread cellular apoptosis, particularly in the central region of the cartilage growth plate [ 2, 10] . Moreover, the individual or combined knockout of *Hif1a* and *Hif2a* reduces EPO expression in chondrocytes and fetal growth plates under both normal and low-oxygen conditions [2]. Intriguingly, the deletion or inhibition of HIF-1α and/or HIF-2α unexpectedly upregulates the expression of hypoxia-induced HBA and HBB. These findings suggest that hypoxia stimulates Hb expression in chondrocytes independently of the HIF signaling pathway.

Krüppel-like factor 1 (KLF1), a critical transcription factor highly expressed in RBCs, regulates the erythropoietic process by controlling the cell cycle to ensure timely differentiation and maturation of RBCs. It also plays a vital role in coordinating the oxygen-sensing mechanism by interacting with EPO and the HIF pathway. KLF1 is essential for the globin switching process, and KLF1 deficiency leads to β-thalassemia [11]. Recent research has identified KLF1 as a potential target of hypoxia, facilitating hypoxia-induced HBA and HBB expressions in chondrocytes by directly interacting with the enhancer and promoter regions at the locus control regions of *Hba* and *Hbb* ( Figure 1) [2]. Knockout of *Hif1a* and *Hif2a* leads to significant upregulation of *Klf1* under various conditions. In addition, hypoxia-mediated inactivation of lysine-specific demethylase 5A (KDM5A), a member of the H3K4me3 demethylase family, suppresses its conventional demethylation activity, thereby epigenetically orchestrating the upregulation of *Klf1* expression through increased H3K4me3 modifications ( Figure 1) [2]. Consequently, Hb production in chondrocytes is regulated by hypoxia and relies on KLF1, which is distinct from the HIF pathway.

Hedy, which functions as a localized oxygen reservoir, plays a crucial role in enabling chondrocyte adaptation to hypoxia and ensuring cartilage survival. The homozygous deletion of *Hba* or *Hbb* in mice results in a slight delay in cartilage hypertrophy, a reduction in pattern defects, and a significant increase in cellular apoptosis in the growth plate region, ultimately leading to embryonic dehydration and mortality [ 2, 7] . Conditional deletion of Hb in cartilage tissue results in structural impairment in Hedy, accompanied by pronounced hypoxia, increased glycolysis, and extensive cell death within the central region of the cartilage tissue [2]. Furthermore, assessment of oxygen dissociation curves revealed a significant left shift in chondrocytes with Hedy syndrome compared with those with erythrocytes, indicating a short-range oxygen supply to the cartilage tissue under hypoxic conditions [2]. In short, Hedy exhibits enhanced oxygen affinity, allowing it to store oxygen in oxygen-deficient environments and supply oxygen in situations of worsened oxygen deficiency or increased oxygen demand. This mechanism facilitates hypoxia adaptation and the survival of chondrocytes.

The viability of cartilage, which lacks a direct blood supply, depends on oxygen levels in its surrounding environment. Hb within chondrocytes potentially contributes to the pathogenesis of various cartilage-related disorders. Patients with thalassemia syndromes often experience joint pain, possibly due to factors such as joint tissue hypoxia from anemia, immune responses triggered by abnormal Hb synthesis, and chronic anemia [12]. Moreover, anemia is prevalent in individuals with cartilage-related diseases. In patients with rheumatoid arthritis (RA), anemia exacerbates cartilage tissue hypoxia by reducing oxygen delivery capacity, leading to increased joint pain. RA patients with higher disease activity tend to have lower hemoglobin levels, indicating more severe joint disease [13]. In individuals with cartilage developmental disorders, reduced Hb adversely affects the oxygen supply during cartilage development, disrupting normal cartilage cell differentiation and extracellular matrix synthesis. Recent studies have indicated that treating anemia to increase Hb levels not only enhances the oxygen supply to body tissues but also significantly benefits joint tissues in conditions related to cartilage disorders [ 14, 15] . The proactive management of anemia could emerge as an effective strategy for treating cartilage-related diseases, offering a promising therapeutic approach to improve joint symptoms.

The mechanism by which nonvascular tissues adapt to hypoxic environments remains unclear. Recent studies have identified a novel Hb condensate named “Hedy” in chondrocytes that displays a granular distribution similar to that observed in the retinal epithelium and in glaucoma. This condensed Hb body stores an increased amount of oxygen in confined spaces, supporting the prolonged oxygen needs of cells in highly oxygen-consuming or avascular tissues. Hypoxia triggers the expression of Hb in chondrocytes, and its depletion results in increased hypoxia, increased glycolysis, and subsequent activation of the HIF signaling pathway.

This article presents a comprehensive overview of the intricate relationship between cartilage tissue and Hb, highlighting the importance of Hb in hypoxic environments and its critical role in cartilage tissue adaptive mechanisms. This exploration contributes to understanding the mechanisms underlying Hb in cartilage-related diseases and advancements in cartilage tissue regeneration and disease treatment. As a novel biomarker in cartilage tissue, Hedy is a promising therapeutic target for pharmacological interventions. Regulation of the expression or function of Hedy may alter the hypoxia adaptation process within cartilage tissue, providing new avenues for the treatment of cartilage-related disorders. The discovery of Hedy provides new research directions for advancements in cartilage tissue engineering as well as regenerative medicine. The ability to design novel biomaterials or construct artificial cartilage tissue to achieve the storage and release of oxygen and other bioactive molecules will result in new strategies and methods for cartilage repair and regeneration. It is possible that the growth and repair of cartilage tissue could be precisely controlled by modulating the content, distribution, and structure of Hedy in relevant biomaterials. Furthermore, we anticipate a thorough understanding of the structural characteristics, regulatory mechanisms, and interactions of Hedy with other cellular organelles within chondrocytes, aiming to reveal the mysterious aspects of Hb in chondrocytes and provide precise targets and new perspectives for future therapeutic strategies.
